# Ovarian hydatid cyst: an uncommon site of presentation

**DOI:** 10.4322/acr.2023.461

**Published:** 2023-12-15

**Authors:** Kaushlendra Kumar, Ariba Zaidi, Nuzhat Husain

**Affiliations:** 1 Dr. Ram Manohar Lohia Institute of Medical Sciences, Department of Pathology, Lucknow, Uttar Pradesh, India

**Keywords:** Echinococcosis, Ovarian cysts, Ovarian neoplasms

## Abstract

Hydatid cyst is a parasitic infestation caused by Echinococcus larvae. Hydatid cyst of the ovary is a highly unusual presentation. Herein, we present a case of a young woman who complained of episodic lower abdominal pain. Ultrasound of the abdomen revealed a multi-cystic left adnexal mass measuring 86 mm x 67 mm. A possibility of ovarian cystic neoplasm was suggested. Unilateral salpingo-oophorectomy was performed. On histopathological examination, a cyst measuring 8.0 x 5.5 x 4.5 cm was found, replacing the entire ovary. The cyst cavity was filled with serous fluid and multiple pearly white membranous structures, giving a multiloculated appearance. Microscopic examination showed a cyst lined by a lamellar membrane containing protoscolices and hooklets. Hydatid disease is a zoonotic ailment caused by tapeworms *(Echinococcus granulosus* or, less commonly, *Echinococcus multilocularis)*. The definitive hosts are carnivores. Humans are the accidental intermediate hosts. The hydatid cyst commonly affects the liver and the lungs. The primary hydatid cyst of the ovary is quite rare, with few case reports in the literature. In most cases, symptoms are vague, and the lesion is misdiagnosed as benign or malignant ovarian cystic neoplasm on clinical and radiological examination. Ovarian hydatid cyst is treated by surgery with ovarian cystectomy as the gold standard. The possibility of a hydatid cyst should be kept under differential diagnoses while evaluating the cystic diseases of the ovary.

## INTRODUCTION

Hydatid cyst is a parasitic infestation caused by Echinococcus larvae, the most common being Echinococcus granulosus. The hydatid cyst commonly affects the lungs and liver. Other locations where hydatid cysts can develop are the kidney, brain, and spleen. Very rarely, this entity is seen in the female reproductive system. In the female genital tract, ovary and uterus are the most commonly affected organs.^1^

Hydatid cyst of the ovary is an extremely unusual presentation of this disease, accounting for about 0.2-1% of all identified cases. Usually, symptoms are vague, and the lesion is misdiagnosed as benign or malignant ovarian cystic neoplasm on clinical and radiological examination.^2^

Herein, we describe one rare case of a hydatid cyst involving the ovary and fallopian tube, diagnosed as an ovarian cyst on ultrasonography.

## CASE REPORT

A 35-year-old woman complained of episodic lower abdominal pain over 2 months. The pain was poorly localized and did not interfere with the patient's daily activities. There was no pain radiation and no associated symptoms. She was having regular menstrual cycles. There was no history of dysmenorrhoea, menorrhagia, hirsutism, weight loss, or gain. There was no significant past or family history. On examination, the patient was not pale or jaundiced, and her vital signs were normal. Abdominal examination elicited deep tenderness in the supra-pubic region. No organomegaly or any palpable mass was noted. There was no fever or any respiratory symptoms. Other systemic examinations were within normal limits.

Routine hematological and biochemical investigation, including hemogram, urine examination, renal and liver function tests, revealed no abnormality.

**Figure 1:**
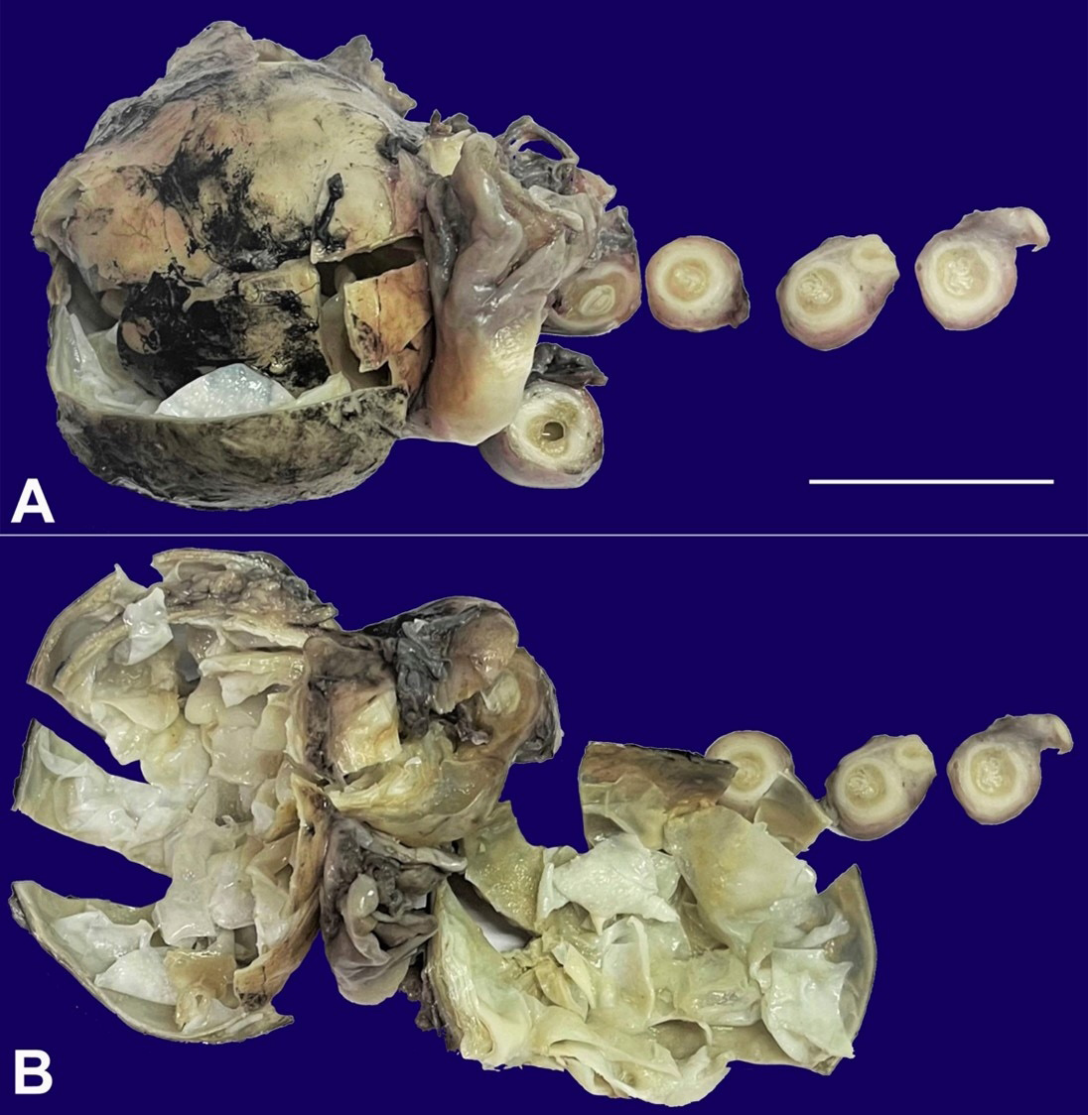
**A** and **B –** Gross view of unilateral salpingo-oophorectomy specimen, showing complete replacement of ovarian parenchyma by a cyst, containing pearly white membranous structures. Transverse sections of the fallopian tube also show similar membranous material (scale bar= 5cm).

The abdominal ultrasonography showed evidence of a polycystic left adnexal lesion measuring 86 mm by 67 mm. No other lesion was found in the abdomen. Tumor markers were not done in this patient. A diagnosis of ovarian cystic disease was suggested based on radiological findings. Unilateral salpingo-oophorectomy was performed, and the specimen was sent to the Pathology Department. On gross examination, the ovarian parenchyma was replaced by a cyst measuring 8.0 x 5.5 x 4.5 cm. The cyst cavity was filled with serous fluid and multiple pearly white membranous structures, giving a multiloculated appearance. There were no papillary excrescences. The Fallopian tube was grossly dilated and filled with similar membranous material (Figure 1).

**Figure 2:**
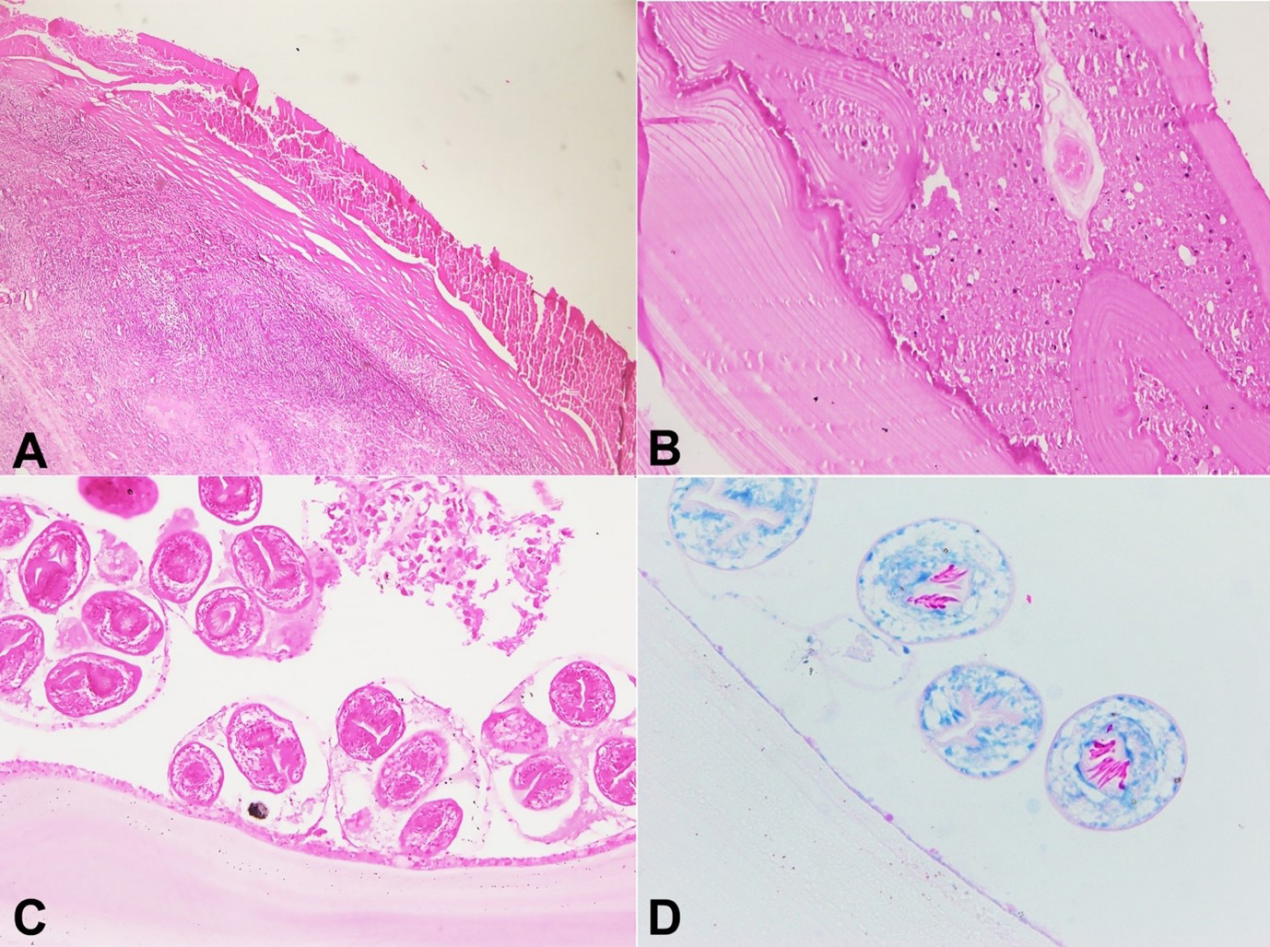
Photomicrographs of the Cyst. **A –** shows a cyst, lined by thin glassy membranous structures with compressed ovarian parenchyma (H&E,100X); **B –** shows a lamellated structure of hyaline membrane along with eosinophilic granular material (Hydatid sand), (H&E, 200X); **C –** shows germinal layer with protoscolices and hooklets, (H&E,400X); **D –** shows protoscolices with hooklets which are acid-fast, (Ziehl-Nielsen, 400X).

On microscopic examination, sections show a cyst lined by a lamellar membrane containing protoscolices and hooklets. (Figure 2A-2C). Hooklets were better highlighted on the Ziehl-Neelsen staining as acid-fast structures. (Figure 2D) Compressed ovarian parenchyma was noted at the periphery. (Figure 2A) Sections from the Fallopian tube show similar findings. Histopathology confirmed the diagnosis of a hydatid cyst involving the Ovary and Fallopian tube.

## METHODS

We searched articles in PubMed, Scopus, and Google Scholar. The terms used for the search were “hydatid cyst of the ovary”, “hydatid cyst in the female genital tract”, and “ovarian echinococcosis”. The search was performed between 5^th^ September 2023 and 8^th^ September 2023. We excluded articles not in English and articles that reported hydatid cysts at sites other than the female genital tract. 39 articles were reviewed by two pathologists (AZ and KK) to avoid duplication. Information about age, site of presentation, clinical features and radiological findings were extracted.

## RESULTS

In the 39 articles reviewed, 42 cases of hydatid cysts in the female genital tract were found. The age range was wide, between 8years to 72years. The ovary was the most common site of involvement of the female genital tract (20/42), followed by the uterus (6/42), broad ligament(4/42), and fallopian tubes(3/42). 5/42 cases were reported as adnexal masses where the exact location of the cyst (ovary/fallopian tube) was not identified. 2/42 cases were reported as pelvic masses. 1/42 was a rare case of paravaginal hydatid cyst. 5/42 cases were of multifocal hydatid cysts involving abdominal organs such as the liver, spleen, and omentum, in addition to the female genital tract. The most common presenting symptom was abdominal pain (31/42) followed by mass per abdomen, abdominal distension, bloating, heaviness in the abdomen, difficulty in micturition, dyspareunia, and fever. 1/42 was incidentally diagnosed as a hydatid cyst of the ovary. All these cases had undergone radiological imaging through ultrasonography, CT (Computed Tomography) scan, or MRI (Magnetic Resonance Imaging). Radiological findings ranged from uniloculated to multiloculated cystic and solid cystic lesions. Some cases were reported as heterogenous (on USG) or hypoechoic masses (CT-Scan). Some of the cysts revealed septations on radiological imaging. None of the features were specific for diagnosing hydatid cysts and were reported as cystic lesions of the ovary or other parts of the genital tracts.

## DISCUSSION

Hydatid disease is a zoonotic ailment. It is caused by tapeworms’ adult or larval stages (*Echinococcus granulosus* or, less commonly *Echinococcus multilocularis)*. The definitive hosts are carnivores like dogs. The parasite’s eggs are excreted in the feces of carnivores and then ingested by herbivores (intermediate hosts) like sheep and cattle. The parasite’s larvae then pass from the intestine of the herbivores to other parts of the body via blood circulation. Humans are the accidental intermediate hosts.^2^ Hydatid cyst commonly affects the liver and the lungs. The primary hydatid cyst of the ovary is quite rare. We could retrieve 42 cases of hydatid cysts in the female genital tract in the literature.^3-6^ These are listed in Table 1.

**Table 1:** Cases of hydatid cyst in female genital tract reported in literature

**ref**	**Age(y)/** **Sex**	**Site**	**Imaging**	**Presentation**
^7^	43/F	O	USG - Multivesicular, fluid-containing cystic lesion in the left adnexa	Left lower quadrant abdominal pain
^8^	43/F	AR	USG - Right adnexal mass	Episodes of ballottement in lower abdomen
	34/F	O	USG - Cystic lesion of left ovary	pain in abdomen and fever
^9^	31/F	AR	CT scan - A large solid cystic mass in left ovary with mild enhancement in the solid sections favoring cystadenoma/adenocarcinoma	Abdominal colic pain in right lower quadrant
	32/F	AR	USG - Paratubal cyst	Pelvic pain and infertility
	23/F	AR	USG - multiple cystic lesions in the right adnexa and ovary	pain in RLQ during pregnancy
^1^	37/F	O	USG - A large heterogenous mass in right ovary	abdominal pain and spotting
^10^	18/F	O	USG - Cystic left adnexal lesion which was univesicular and contained fluid CT Scan - Cystic left adnexal lesion with no enhancement	Episodes of lower abdominal pain and frequent urination
^11^	62/F	U	USG - Uterine fundal mass ELISA – Positive	Chronic pelvic pain
^12^	45/F	FT	CT Scan - Well defined hypodense pelvic cystic lesion with multiple daughter cysts consistent with hydatid disease	Painless lower abdomen lump
^13^	72/F	O	USG - Cystic multilocular tumor with solid components and presence of acoustic shadow under the right adnexa,	Uterine prolapse
^14^	62/F	U	USG - A large pelvic and cystic mass	Lower abdominal pain
^15^	42/F	FT	USG - A multiloculated cystic lesion of 32 × 35 mm in the left adnexa	Lower abdominal pain
^5^	37/F	O	CT Scan - A large multiseptated cystic lesion in the pouch of Douglas	increasing abdominal girth, weight loss, fever
^16^	71/F	U+ colon	MRI - Type 3 hydatid cyst with daughter vesicles located at the posterior of uterus and type 2 hydatid cyst with detached membrane in the lesion	Chronic abdominal, pelvic pain and abdominal swelling
^17^	44/F	O + om	A large mass with branched septations and solid components	Abdominal distension
^18^	46/F	U	USG - A cyst showing internal septations and some daughter cysts and some echogenic debris CT Scan - Multiloculated cyst with a thick wall on left side of the PR	Pelvic pain, nausea and anorexia
^19^	30/F	O	USG - Bilateral ovarian masses with hemorrhagic cyst in one ovary and cyst in the other ovary	Lower abdominal pain
^20^	24/F	Liver and O	USG - Large thick-walled, multiloculated cystic lesion with thick septations in the right adnexa with non-visualization of the right ovary separately and a small thick-walled unilocular cyst seen in the left ovary MRI - A large circumscribed cystic lesion with septations in the right adnexa Another cystic lesion was seen in the left ovary	Pelvic discomfort
^21^	22/F	BL	USG - A large pelvic and lower abdominal multi-septated cystic mass MRI - A large multi-loculated cystic mass, occupying the whole pelvis and extending into the left lower abdominal quadrant	Constipation and hematuria with acute urinary retention
^22^	66/F	O	USG - Cystic mass with echogenic elements localized in the anatomical site of the left ovary	Fever, abdominal pain and discomfort
^23^	27/F	Cervix	USG - An anechoic cystic mass with thin wall of 4 mm, located at the posterior wall of the cervical canal towards to the Douglas pouch	Dyspareunia and chronic pelvic pain
^24^	69/F	FT	USG - A multiloculated mass with thick septation in the right adnexa	Abdominal pain and urinary frequency
^25^	8/F	O+BL	USG - A multilocular cystic lesion, with multiple internal septations and no calcifications in the right adnexa with a unilocular lesion in the left adnexa CT Scan - A multilocular lesion in the right ovary and a unilocular cystic lesion in the left adnexa	Recurrent lower abdominal pain
^26^	35/F	BL	USG - Right sided ovarian cystic mass without any septation CT Scan - a hypoechoic mass in pelvic origin.	Difficulty in micturition, abdominal heaviness and enlarging abdominal lump
^27^	19/F	Abd, spl, O	USG - Thin-walled septated cysts in the left ovary and thin-walled multiloculated cysts in the spleen	Abdominal pain
^28^	66/F	U	USG - A 10x7 cm sized cystic lesion of the uterus CT Scan - A multiseptated cystic lesion of the uterus	Tenesmus and lower abdominal pain
^29^	12/F	O	USG - A cystic lesion of ovary, suggestive of ovarian tumor CT Scan - Multiseptated cystic lesion of right ovary	Abdominal pain and complaints of urinary obstruction
^30^	38/F	O	MRI - Cystic lesion in the right ovary surrounded by loculated fluid collection with septations	Right lower abdominal pain
^6^	30/F	O	USG - A solid-to-cystic mass lesion	pain in the lower abdomen with abdominal distension
^31^	58/F	PR	USG - A huge mass within the umbilical region, and another mass arising from the right pelvis	Abdominal swelling, gastric complaints, and pelvic pain
^32^	76/F	O	CT Scan - Hepatic hydatid cyst and a multiloculated cyst situated in the pouch of Douglas	Pain in the lower abdomen
^2^	30/F	O	USG - A huge multiloculated mass occupying the pelvis with internal debris	Amenorrhea, intermittent bleeding P/V and an abdominal mass
^33^	43/F	PV	USG - A heterogeneous hypoechoic mass located in the pelvis, containing multiple anechoic small nodules. MRI - Round-shaped hyperintense mass lesion at the right paravesical, paravaginal space	Pelvic pain, frequent urination, and dyspareunia
^34^	27/F	O	USG - A multicystic septate mass in the right adnexa with solid areas	Abdominal pain, backache, and menorrhagia
^35^	28/F	AR	**USG** - A hypoechogenic multiloculated cystic mass in the left ovarian location	Left adnexal mass on routine examination
^3^	64/F	O	USG - A well- defined large multilocular cyst localized in right adnexal region	Chronic pelvic pain and abdominal discomfort
^4^	76/F	O	USG - A round, heterogeneous mass with solid and cystic components located in the pelvis. CT Scan - Multiloculated, heterogeneous mass lesion in the pelvic cavity	Urinary retention
^36^	25/F	PR	USG - Well-defined, multicystic, hypoechogenic mass with solid components	Chronic pelvic and minor epigastric discomfort
^37^	70/F	U	USG - A cystic area with regular borders in the uterus CT Scan - Radiolucent mass with well-circumscribed borders in the cavity of the uterus	Lower abdominal pain
^38^	27/F	O	USG - Hypoechogenic cyst with internal echoes in the right ovary	Incidental
^39^	34/F	BL		increasing lump in the lower abdomen

Abd = abdomen, AR = adnexal region, BL = broad ligament, FT = Fallopian tube, F = female, M = male, Om = omentum, O = ovary, Ref = reference, y = year, RLQ = right lower quadrant, PR = pelvic region, Spl = spleen, U= uterus.

The age of presentation of Hydatid cyst in the female genital tract ranged between 8 and 72 years. In the various cases reported in the literature, the ovary is the most common site in the female genital tract, followed by the uterus, broad ligament, and fallopian tubes. Ovarian hydatidosis usually presents with non-specific symptoms. The most common symptom is abdominal or pelvic pain, followed by mass per abdomen. (Table 1) Ultrasound is an important imaging modality for detecting hydatid disease. Hydatid cyst has a cystic to solid appearance on ultrasonography. A Computed Tomography (CT) scan with a higher sensitivity is considered a superior imaging modality. The radiological features of various cases of hydatid cysts in the female genital tract, reported in the literature, have been described as ranging between cystic to solid-cystic lesions with or without septations.(Table 1) However, no definite features have been described specific to the diagnosis of Hydatid cyst. Only a few case reports were found where the diagnosis of a hydatid cyst was suggested on either Magnetic Resonance Imaging (MRI) or Computed Tomography (CT) scan. (Table 1) Serological tests are very useful for confirming the diagnosis with a 60-90% sensitivity. These include indirect hemagglutination (IHA), immunoblotting, enzyme-linked immunosorbent assay (ELISA), indirect fluorescent-antibody (IFA), latex agglutination test, and immunochromatography tests. Screening tests such as Enzyme immunoassay or indirect hemagglutination and confirmatory tests such as immunoblot or Gel diffusion are available for diagnosing this disease. However, cysticercosis may cause false positive reactions with these tests.^2^ Fine needle aspiration cytology (FNAC) also helps establish the diagnosis, although this technique carries a risk of anaphylactic reaction due to spillage of cyst fluid.^6^ Diagnosis of Hydatid cyst can be confirmed on histopathological examination of the excised cyst. Microscopic evaluation of the hydatid cyst shows three layers of cyst wall. The innermost germinal layer has a thin and translucent appearance. In the embryonic tapeworm, scolices develop from an outpouching of the germinal layer and form hydatid sand. The middle laminated membrane is white, approximately 2 mm thick. The outer layer or pericyst is a rigid protective layer of granulation tissue and fibrosis, representing the host’s response to the parasite. Our case showed gross and microscopic findings consistent with hydatid cysts in form of scolices, an acellular, thick lamellar membrane, and a surrounding pericyst. Ovarian parenchyma was compressed and seen at the periphery of the cyst.

Asymptomatic small, unilocular cysts are treated with anthelminthic drugs such as Albendazole, but large and symptomatic cysts should be treated surgically. Other alternatives include PAIR therapy, in which ultrasound-guided percutaneous aspiration of cysts is done. This is followed by injecting protoscolicidal agents such as 20% NaCl solution, 95% ethanol, 50% glucose, or silver nitrate. Then, the cyst content is re-aspirated after 15 minutes of contact period. This should be avoided in lung cysts and communicating cysts.^2^

Ovarian hydatid cyst is treated by surgery, which could be radical or conservative, with ovarian cystectomy as the gold standard. Surgery is done to remove all the cysts and prevent cyst contents’ spillage.^32^

## CONCLUSION

Ovarian Hydatid cyst mimics either a polycystic ovarian disease or solid cystic neoplasms of the ovary. The symptoms and radiological features are also not specific. Owing to its multilocular cystic appearance, an ovarian hydatid cyst may resemble the cyst septations and thus raise suspicion of cystic neoplasms of the ovary. This leads to unnecessary anxiety for the patient. Hence, gynecologists, radiologists, and pathologists should be aware of this entity in the ovary (Figure 1). A rare possibility of a hydatid cyst should be kept under differential diagnoses while evaluating the cystic diseases of the ovary.
